# Antifungal Activity, Toxicity, and Membranolytic Action of a Mastoparan Analog Peptide

**DOI:** 10.3389/fcimb.2019.00419

**Published:** 2019-12-06

**Authors:** Junya de Lacorte Singulani, Mariana Cristina Galeane, Marina Dorisse Ramos, Paulo César Gomes, Claudia Tavares dos Santos, Bibiana Monson de Souza, Mario Sergio Palma, Ana Marisa Fusco Almeida, Maria José Soares Mendes Giannini

**Affiliations:** ^1^Department of Clinical Analysis, School of Pharmaceutical Sciences, São Paulo State University-UNESP, Araraquara, Brazil; ^2^Department of Biology, Center for the Study of Social Insects, Institute of Biosciences, São Paulo State University-UNESP, Rio Claro, Brazil

**Keywords:** invasive fungal infections, antifungal, cell membrane, invertebrate models, antimicrobial peptide

## Abstract

Invasive fungal infections, such as cryptococcosis and paracoccidioidomycosis are associated with significant rates of morbidity and mortality. Cryptococcosis, caused by *Cryptococcus neoformans*, is distributed worldwide and has received much attention as a common complication in patients with HIV. Invasive fungal infections are usually treated with a combination of amphotericin B and azoles. In addition, 5-fluorocytosine (5-FC) is applied in cryptococcosis, specifically to treat central nervous system infection. However, host toxicity, high cost, emerging number of resistant strains, and difficulty in developing new selective antifungals pose challenges. The need for new antifungals has therefore prompted a screen for inhibitory peptides, which have multiple mechanisms of action. The honeycomb moth *Galleria mellonella* has been widely used as a model system for evaluating efficacy of antifungal agents. In this study, a peptide analog from the mastoparan class of wasps (MK58911) was tested against *Cryptococcus* spp. and *Paracoccidioides* spp. In addition, peptide toxicity tests on lung fibroblasts (MRC5) and glioblastoma cells (U87) were performed. Subsequent tests related to drug interaction and mechanism of action were also performed, and efficacy and toxicity of the peptide were evaluated *in vivo* using the *G. mellonella* model. Our results reveal promising activity of the peptide, with an MIC in the range of 7.8–31.2 μg/mL, and low toxicity in MRC and U87 cells (IC_50_ > 500 μg/mL). Taken together, these results demonstrate that MK58911 is highly toxic in fungal cells, but not mammalian cells (SI > 16). The mechanism of toxicity involved disruption of the plasma membrane, leading to death of the fungus mainly by necrosis. In addition, no interaction with the drugs amphotericin B and fluconazole was found either *in vitro* or *in vivo*. Finally, the peptide showed no toxic effects on *G. mellonella*, and significantly enhanced survival rates of larvae infected with *C. neoformans*. Although not statistically significant, treatment of larvae with all doses of MK58911 showed a similar trend in decreasing the fungal burden of larvae. These effects were independent of any immunomodulatory activity. Overall, these results present a peptide with potential for use as a new antifungal drug to treat systemic mycoses.

## Introduction

Infections caused by fungi have increased dramatically in recent decades, and are associated with significant rates of morbidity and mortality (Thompson et al., 2016). In addition, an increase in the number of immunocompromised patients (transplant recipients, individuals infected with human immunodeficiency virus (HIV), and individuals with cancer), as well as geoclimatic changes, have led to more serious fungal infections (Brown et al., 2012; Armstrong-James et al., 2014). In this context, the systemic mycoses caused by *Cryptococcus* spp. and by dimorphic fungi, such as *Paracoccidioides* spp. have been recognized as a growing problem for human health (Thompson et al., 2016).

*Cryptococcus neoformans* and *C. gattii* are two etiological agents of cryptococcosis, estimated to be responsible for 278,000 of infections and 181,000 deaths annually worldwide. Cryptococcosis in particular, which is caused by *C. neoformans* and has a worldwide distribution, has received great attention as a common complication in patients with HIV (Rajasingham et al., 2017). Additionally, *Paracocciodioides brasiliensis* and *P. lutzii* are responsible for paracoccidioidomycosis, which is an endemic mycosis in South and Central America (Shikanai-Yasuda et al., 2006; Arantes et al., 2016).

Treatment of mild to moderate cryptococcosis is usually done with the administration of fluconazole and severe cases including cryptococcal meningitis, are treated with amphotericin B combined with 5-fluorocytosine (5-FC) or fluconazole (Mourad and Perfect, 2018). On the other hand, patients with the paracoccidiodomycosis are more commonly treated with amphotericin B, itraconazole and cotrimoxazole (sulfamethoxazole/trimethoprim combination) (Shikanai-Yasuda et al., 2017). Recent strategies, such as immunotherapy (interleukins, peptide P10 and monoclonal antibodies against virulence factors), new compounds (APX001, AR-12, T-2307, VT1129, VT1598, and natural products) and repurposing drugs (sertraline, tamoxifen, and tacrolimus) have been evaluated in *Cryptococcus* and *Paracoccidioides* infections (de Oliveira et al., 2015; Mourad and Perfect, 2018). Amphotericin B and azoles act on the fungal cell membrane, either by disrupting it or by inhibiting sterol synthesis, whereas 5-fluorocytosine (5-FC) inhibits the nucleic acid synthesis of fungal cell (Scorzoni et al., 2017). However, high systemic toxicity and development of resistance are major problems associated with the clinical use of amphotericin B, azoles, and 5-FC.

In addition, because fungal cells are eukaryotic, developing selective antifungal agents is difficult (Perfect, 2017). Consequently, there has been a marked decline in the development of new antifungals, posing a great challenge to modern medicine. The last antifungals developed were the echinocandins, which function by inhibiting glucan synthesis in the cell wall (Prasad et al., 2016; Scorzoni et al., 2017). However, there is an increasing number of resistant isolates also to this drug class, which can occur many ways, including mutations of three genes FKS1, FKS2, and FKS3. These mutations result in amino acid substitutions and consequently, in different phenotypes of glucan synthase that alter the drug-target interactions (Garcia-Effron et al., 2009; Perlin, 2015). Moreover, *C. neoformans* is intrinsically resistant to echinocandins and the efficacy of these antifungal agents is questionable against dimorphic fungi (Denning, 2002). Thus, there is a great need to develop new classes of antifungal agents with good action against these fungi and minimal side effects. In this context, the identification of antifungal peptides can be one of the useful strategies for this task.

Antimicrobial peptides (AMPs) are important molecules found in nature. These components act as a first line of defense to combat pathogens, and have extensive biological activities, such as anti-inflammatory properties and a broad spectrum of activity against Gram-positive and Gram-negative bacteria, fungi, viruses, and tumor cells (da Costa et al., 2015; Wang et al., 2016). AMPs are unique in that they have multiple targets and mechanisms of action, which is useful for decreasing the emerging threat of multidrug-resistant strains (Zaiou, 2007; Yount and Yeaman, 2012; Haney et al., 2017; Kang et al., 2017).

AMPs of the mastoparan class are isolated from social wasp venom and are composed of polycationic peptides with 14 amino acid residues and an amidated C-terminus. They contain 2–4 lysine residues, and have a positive net charge and an alpha-helical conformation (Rangel et al., 2011; Silva et al., 2017). Some mastoparan peptides have been well-studied and are known to possess antifungal activity (Silva et al., 2017). These mastoparan AMPs may act by altering membrane permeability or by directly forming pores in the membrane (Choi and Lee, 2014; Rautenbach et al., 2016).

Several methods have been tested for evaluating efficacy and safety of new drugs, including antifungals. In order to avoid or replace animal experiments, mini-hosts have emerged as an alternative system for the study of fungal diseases, and for testing chemical libraries to discover new antifungals. These organisms are more ethically suitable, less expensive, and easier to use compared to mammals. The use of invertebrate animals, such as the honeycomb moth *Galleria mellonella*, is increasing, especially in the study of systemic mycoses (Kavanagh and Sheehan, 2018; Singulani et al., 2018).

*G. mellonella* is a moth that feeds on honey, pollen, and beeswax. The viability of its larvae can easily be verified by observing movement and melanization induced in response to infection (Fuchs et al., 2010; Desalermos et al., 2012). The virulence of different fungi, including *Paracoccidioides* and *Cryptococcus* species (Mylonakis et al., 2005; Thomaz et al., 2013; Scorzoni et al., 2015; de Lacorte Singulani et al., 2016; Sangalli-Leite et al., 2016; Palanco et al., 2017) has been successfully studied in *G. mellonella*. In addition, the effect of azoles and amphotericin B, as well as natural products, have been evaluated in this model (Scorzoni et al., 2013; Favre-Godal et al., 2014; de Lacorte Singulani et al., 2016).

The aim of the present study was to evaluate the *in vitro* antifungal activity of a mastoparan analog against *Cryptococcus* and *Paracoccidioides* species, as well as to determine its toxicity on lung and neural cells. In addition, the mechanism of action and *in vivo* potency of the peptide were evaluated through flow cytometry assays and in the *G. mellonella* model, respectively.

## Materials and Methods

### Fungi

Fungal strains used in this study were *C. neoformans* ATCC 90112*, C. gattii* ATCC 56990*, P. brasiliensis* S1 isolate Pb18 (Pb18-chronic PCM/São Paulo, Brazil), and *P. lutzii* Pb01-like strain ATCC MYA-826 (acute PCM/Goiânia, Brazil), which was obtained from the collection of the Laboratory of Clinical Mycology, Faculty of Pharmaceutical Sciences of UNESP, Araraquara (Brazil). *Cryptococcus* species were maintained in Sabouraud dextrose agar at 30°C for 48 h and *Paracoccidioides* species were maintained in Fava-Netto agar at 37°C for 4 days. Biosafety Level 2 protocols were used in experiments with these fungi.

### Peptide

The mastoparan analog peptide (MK58911) used in this study was previously engineered and synthesized according to Souza et al. (2015). The number 58911 in its name indicates the position of lysine residues in the sequence INWLKIAKKVKGML-NH_2_. The peptide was solubilized in sterile water to prepare the stock solution.

### Microdilution Susceptibility Test

A microdilution susceptibility test was performed to analyze the minimal inhibitory concentration (MIC) of peptide MK58911, which means the minimum concentration of a compound capable of inhibiting microorganism growth. The assay was conducted according to CLSI M27-A3 method (2008), using an inoculum of 2.5 × 10^3^ cells/mL in RPMI-1640 medium (Sigma-Aldrich) supplemented with 0.03% (w/v) L-glutamine and 2% (w/v) glucose, and buffered to pH 7.0 with 0.165 M 4-morpholinopropanesulfonic acid, and evaluated in 96-well microplates. For *Paracoccidioides* species, some modifications were made according to de Paula E Silva et al. (2013). Serial 2-fold dilutions of the peptide were prepared to obtain final concentrations ranged from 0.48 to 250 μg/mL. Amphotericin B (0.016–8 μg/mL), fluconazole (0.063–32 μg/mL), and itraconazole (0.016–8 μg/mL) were used as controls. For *Cryptococcus* species, the plates were incubated at 37°C with agitation (150 rpm) for 48 h and fungal growth was visually verified. For *Paracoccidioides* species, plates were incubated at 37°C with agitation (150 rpm) for 72 h and fungal growth was determined by measuring absorbance at 570–600 nm using the indicator Alamar Blue (BioSource International). The MIC value was determined for each fungus species. *Candida albicans* ATCC 90028 was used as reference strain for quality control of the susceptibility tests.

### Cytotoxicity Test

Cytotoxic activity was evaluated in an MRC5 cell line (lung fibroblasts) and U87 cell line (glioblastoma), which were obtained from Banco de Células do Rio de Janeiro (BCRJ Federal University of Rio de Janeiro, Brazil). The cells were cultured in Dulbecco's high glucose Modified Eagle Medium (DMEM; Gibco; Thermo Fisher Scientific) supplemented with 10% fetal bovine serum (FBS) and were incubated at 37°C with 5% CO_2_. The experiment was performed using 96-well plates with 5 × 10^4^ cells/well, and cells were incubated at 37°C with 5% CO_2_ for 24 h for monolayer formation to occur. Serial 2-fold dilutions of the peptide were prepared to obtain final concentrations ranged from 0.96 to 500 μg/mL. The plates were incubated at 37°C for 24 h and cell viability was evaluated by measuring absorbance at 570–600 nm using the indicator resazurin at a concentration of 0.01%. The half maximal inhibitory concentration (IC_50_) was determined and the selective index (SI) of the peptide was calculated according to the formula:

$$\begin{array}{l} \text{SI }=\;\frac{\text{I}{\text{C}}_{50}}{\text{MIC}} \end{array}$$

### Checkerboard Test

Two different combinations of peptide and standard drug were tested: MK58911 + amphotericin B and MK58911 + fluconazole. *C. neoformans* was selected for this test and for subsequent experiments (mechanism of action and *Galleria mellonella* model system) because it causes the highest incidence of systemic mycoses worldwide (mainly in HIV patients) compared to other fungi evaluated in this study (Tenor et al., 2015). 2.5 × 10^3^ cells/mL were inoculated and MK58911 concentration ranged from 1.95 to 250 μg/mL. Amphotericin B and fluconazole were diluted in RPMI 1640 (Sigma-Aldrich) using the same concentrations as the susceptibility test. The plates were incubated at 37°C for 48 h and fungal growth was visually verified. Subsequently, the interaction coefficient was evaluated using the fractional inhibitory concentration (FIC) according to formula:

$$\begin{array}{l} \text{FIC }=\;\left(\frac{\text{MIC MK}58911\text{ in combination}}{\text{MIC MK}58911\text{ alone}}\right)+\left(\frac{\text{MIC FLU or AMB in combination}}{\text{MIC FLU or AMB alone}}\right) \end{array}$$

The interaction between the agents was defined as follows: synergistic if FIC ≤0.5; indifferent if FIC >0.5 and ≤4; and antagonistic if FIC >4 (Reichert-Lima et al., 2016).

### Propidium Iodide Influx

One hundred microliters of a suspension of *C. neoformans* cells (1 × 10^6^ cells/mL) in RPMI medium were treated with 100 μL of MK58911 peptide in the following concentrations: MIC, 0.5 × MIC, and 2 × MIC. Amphotericin B and fluconazole were used as controls at MIC. The plate was incubated for 4 h or 24 h at 37°C with constant shaking (150 rpm). Subsequently, propidium iodide (10 μg/mL) was added to wells and the plate was incubated for 5 min at room temperature in the dark. The cells were analyzed by flow cytometry (BD FACSCanto). Ten microliters of calcofluor white (50 μg/mL) were added and images were captured using the automated microscope IN Cell Analyzer 2000 (GE Healthcare).

### Annexin Staining

The exposure of *C. neoformans* to MK58911 was performed as previously described for propidium iodide influx.

### Detection of Reactive Oxygen Species (ROS) by Dichlorofluorescin Diacetate

The exposure of *C. neoformans* to MK58911 was performed as previously described for propidium iodide influx.

### Efficacy and Toxicity in *G. mellonella* Model

Larvae (0.15–0.20 g) without dark spots were selected and maintained in Petri dishes (*n* = 8–10 for each group) in the dark at 37°C the night before experiments. For efficacy assays, larvae were inoculated with 10 μL of 1 × 10^5^ cells/larvae of *C. neoformans* yeast suspension prepared in PBS with 20 mg/L ampicillin. After an hour of infection, larvae were inoculated with 10 μL of MK58911 at a concentration of 10, 50, or 100 mg/Kg, or with only PBS as a control. For experiments of treatment combinations (MK58911 + amphotericin B and MK58911 + fluconazole), 5 μL of each of the solutions were inoculated, resulting in a final dose of 4 mg/Kg for amphotericin B and 10 mg/Kg for MK58911 and fluconazole. Yeast suspensions and treatments were injected in the back left and right pro-leg of the larvae, respectively, which were previously cleaned with 70% alcohol. For toxicity assays, non-infected larvae were injected with the same doses of peptide. In addition, a group of non-infected larvae inoculated with PBS was used as a control in both assays. The viability of larvae was evaluated daily for 5 days.

### Fungal Burden

*G. mellonella* larvae (*n* = 5 for each group) were infected with *C. neoformans* (1 × 10^5^ cells/larvae) and treated with MK58911 at 10, 50, or 100 mg/Kg or amphotericin B at 4 mg/Kg. A group of infected non-treated larvae was used as a control. Larvae were incubated at 37°C for 24 h. Subsequently, larvae from each group were surface sterilized in 70% ethanol, cut into small pieces using a scalpel, and suspended in 1 mL of PBS with 20 mg/L ampicillin. Tissues were transferred to conical tubes with glass beads and homogenized using a vortex mixer (Norte Científica). Each sample was diluted 100× or 10× in PBS, and 100 μL of the resulting dilution was plated on Sabouraud dextrose agar supplemented with 0.1% chloramphenicol. The plates were incubated at 37°C for 48 h and colony-forming units (CFU) were counted.

### Haemocyte Density

*G. mellonella* larvae (*n* = 5 for each group) were injected with MK58911 at 10, 50, or 100 mg/Kg and incubated at 37°C for 4 or 24 h. Larvae inoculated with PBS were used as a control. Subsequently, the hemolymph was collected and diluted 1:20 in cold PBS. The haemocyte were counted using a Neubauer hemocytometer under a brightfield microscope.

### Statistical Analysis

All data in this study are representative of at least three independent experiments with mean ± S.D. Graphs and statistical analyses were performed with GraphPrism 5.0 (GraphPad Software Inc., La Jolla, CA). The data from mechanism of action studies, fungal burden, and haemocyte density were compared using a one-way analysis of variance (ANOVA) followed by Dunnett's test (*P* < 0.05). The data from *in vivo* efficacy studies were plotted as Kaplan–Meier survival curves and compared using log-rank tests.

## Results

### MK58911 Shows *in vitro* Activity Against Fungi Causing Systemic Mycoses and Has Low Toxicity in Mammalian Cells

Our results demonstrated that the peptide MK58911 had activity in the following order: *P. brasiliensis* > *P. lutzii* = *C. gattii* > *C. neoformans*, with MIC values ranging from 7.8 to 31.2 μg/mL (Table 1). In addition, MK58911 was not toxic in lung fibroblasts (MRC5) and glioblastoma cells (U87) at tested concentrations, and a high IC_50_ of >500 μg/mL was observed in both mammalian cells. From these data, the selectivity index (SI), which consists of the ratio between the IC_50_ for mammalian cells and MIC for fungi, was calculated. This index is a measure of the efficacy of a compound against fungus without causing damage to host cells. The IS ranged from >16 for *C. neoformans* to >64 for *P. brasiliensis*.

**Table 1:** Minimal inhibitory concentration (MIC in μg/mL) for *Cryptococcus* and *Paracoccidioides* species, half maximal inhibitory concentration (IC_50_ in μg/mL) in MRC5 cell line (lung fibroblasts) and U87 cell line (glioblastoma), and selectivity index (SI) of MK58911.

	**MK58911**	**Amphotericin B**	**Fluconazole**	**Itraconazole**
*C. neoformans*	31.2	0.12	1	–
*C. gattii*	15.6	0.12	8	–
*P. brasiliensis*	7.8	0.12	–	0.008
*P. lutzii*	15.6	0.25	–	0.008
IC_50_-MRC5	>500	–	–	–
IC_50_-U87	>500	–	–	–
SI (IC_50_/MIC)	>16^*^	–	–	–

* *Related to C. neoformans*.

### MK58911 Did Not Have a Synergic Effect With Antifungal Drugs

The interaction between MK58911 and amphotericin B or fluconazole drugs was evaluated against *C. neoformans* through the checkerboard test. For both combinations, FIC values of >0.5 to ≤4 were found, indicating that the interactions were indifferent.

### MK58911 Causes Membrane Damage on Fungal Cells Through Necrosis and Apoptosis

**Figure 1:**
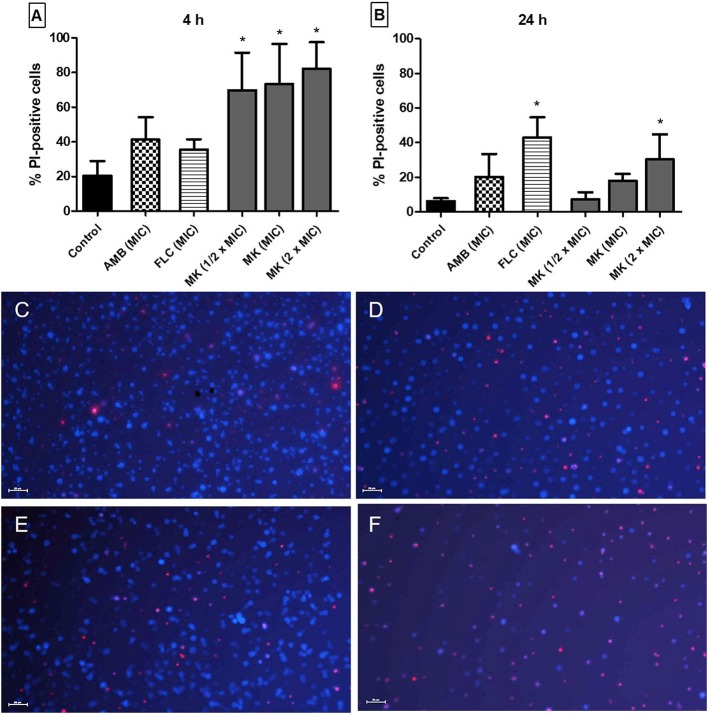
Effect of different concentrations of MK58911 (MK – 1/2 × MIC; MIC and 2 × MIC), amphotericin B (AMB–MIC), or fluconazole (FLC–MIC) on *C. neoformans* membrane, evaluated by percentage of propidium iodide (PI)-positive cells at **(A)** 4 h and **(B)** 24 h. ^*^p < 0.05 compared to control group. Representative images of *C. neoformans* **(C)** non-treated cells or **(D)** cells treated at MIC of amphotericin B **(E)** MIC of fluconazole or **(F)** MIC of MK58911 at 4 h. The cell wall of all fungal cells was stained with calcofluor (blue), and cells with membrane damage were stained with propidium iodide (red). Scale bar: 20 μm.

To test possible cell membrane effects of the peptide, we performed an assay to evaluate membrane integrity through measuring PI influx. *C. neoformans* cells treated with MK58911 internalized more PI in a concentration-dependent manner over the course of the experiment (Figure 1). After 4 h of incubation, all the tested concentrations (1/2 × MIC; MIC and 2 × MIC) caused membrane damage to fungal cells at significantly higher levels (69.9; 73.4 and 82.23, respectively) compared to non-treated cells (20.4; control with *p* < 0.05), and the treatment with amphotericin B at MIC (41.3), followed with fluconazole at MIC (35.5; Figures 1A,C–F). In contrast, after 24 h, MK58911 at 2 × MIC (30.4) and fluconazole (43.0) showed significantly more PI influx compared to the control (6.3; Figure 1B).

**Figure 2:**
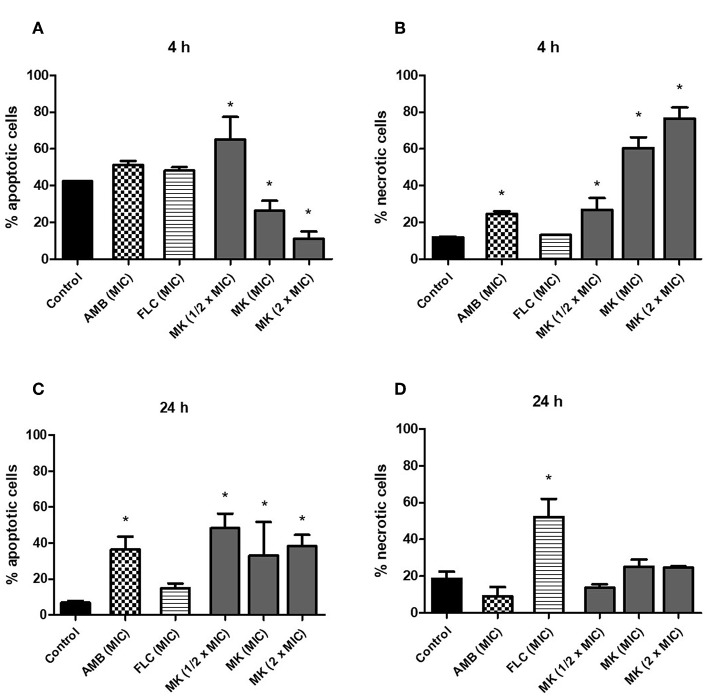
Effect of different concentrations of MK58911 (1/2 × MIC, MIC, and 2 × MIC), amphotericin B (MIC), or fluconazole (MIC) on *C. neoformans* death by apoptosis and necrosis at **(A,B)** 4 h and **(C,D)** 24 h. ^*^p < 0.05 compared to control group.

**Figure 3:**
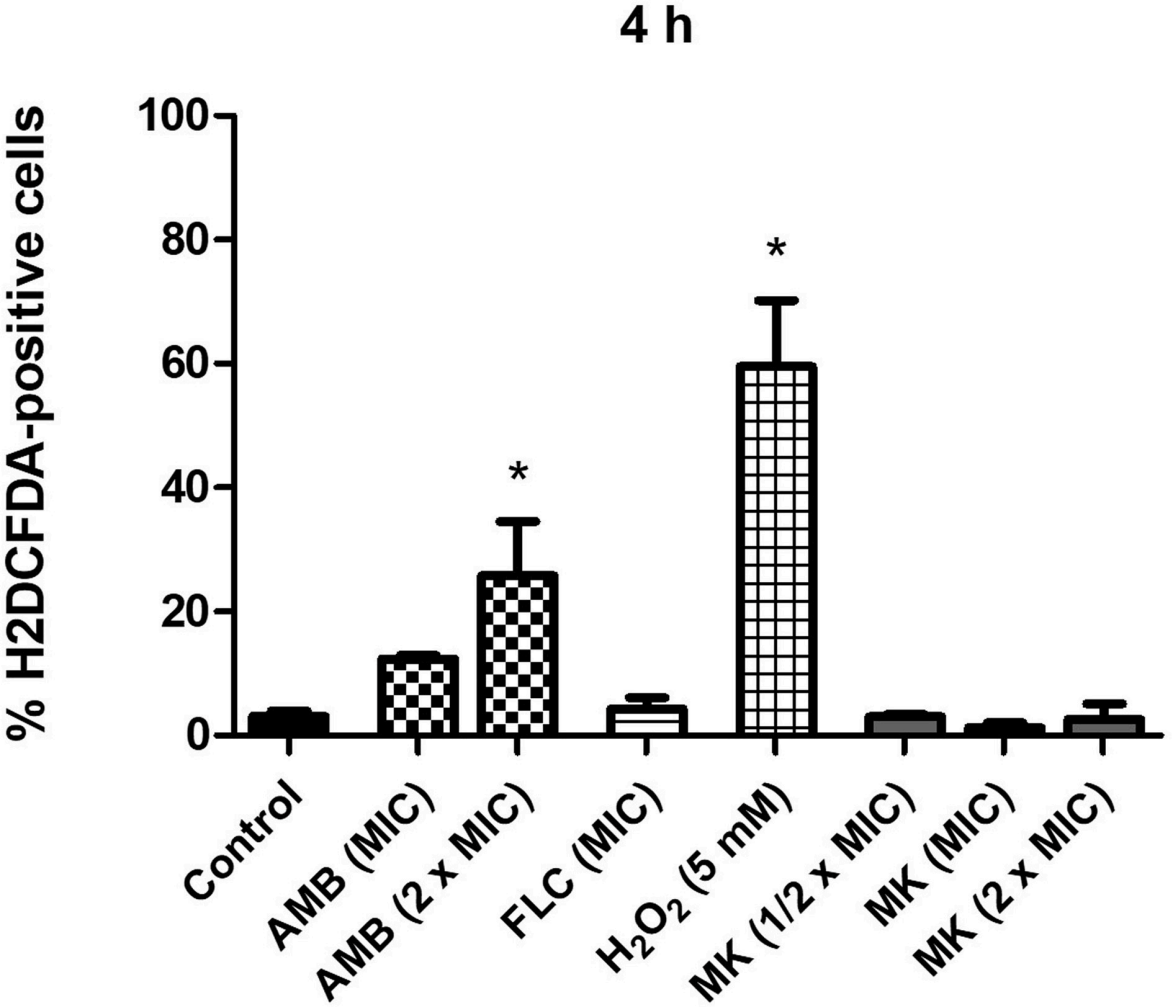
Effect of different concentrations of MK58911 (1/2 × MIC, MIC, and 2 × MIC), amphotericin B (MIC), or fluconazole (MIC) on induction of oxidative stress in *C. neoformans* at 4 h. ^*^p < 0.05 compared to control group.

The effect of MK58911 on *C. neoformans* cell death was also evaluated through measuring apoptosis and necrosis with annexin V and PI staining. After 4 h, the percentage of apoptotic cells was inversely proportional to the peptide concentration, while the percentage of necrotic cells was directly proportional to the peptide concentration (Figures 2A,B; *p* < 0.05 compared to control). In addition, there was an increase in the percentage of necrotic cells treated with amphotericin B (Figure 3B; *p* < 0.05 compared to control), and no significant differences were observed between cells treated with fluconazole and the control (Figures 2A,B). After 24 h, the peptide caused an increase in the percentage of apoptotic cells at all concentrations tested (Figure 2C; *p* < 0.05 compared to control). Cells treated with amphotericin B (MIC) had a higher percentage of apoptotic events (Figure 2C; *p* < 0.05 compared to control), and those treated with fluconazole (MIC) had the most necrotic events (Figure 2D; *p* < 0.05 compared to control).

### MK58911 Does Not Lead to ROS Production in *C. neoformans*

Since action of the peptide was more pronounced at 4 h compared to 24 h, we tested its effect on ROS production in fungal cells at this time point. For this purpose, H2DCFDA was used, which is hydrolyzed by esterases inside of cells to 2′,7′-dichlorofluorescine. When this compound contacts ROS, 2′,7′-dichlorofluorescein (DCF) is generated, which is fluorescent. As shown in Figure 3, the mean fluorescence values (1.7–3.0%) of *C. neoformans* cells were not increased at different concentrations of MK58911 ($\frac{1}{2}$ × MIC, MIC, and 2 × MIC) compared to the control (3.1%), suggesting that the induction of ROS is not a mechanism of action for this peptide. Similarly, ROS production was not observed when fungal cells were exposed to the MIC of fluconazole (4.3%). On the other hand, amphotericin B at MIC and 2 × MIC lead to an increase in ROS levels in a concentration-dependent manner, with mean values of 12.4 and 25.8% (*p* < 0.05 compared to the control), respectively. H_2_O_2_, a compound known to generate ROS, was tested at a concentration of 5 mM, and significantly increased fluorescence (59.5%) of *C. neoformans* cells compared to the control (*p* < 0.05).

### MK58911 Has Antifungal Efficacy and No Toxicity When Tested *in vivo*

In order to evaluate *in vivo* effects of MK58911 on *C. neoformans*, we used a *G. mellonella* animal model. MK58911 at 10, 50, or 100 mg/Kg were administered to non-infected larvae to determine toxicity. No death and melanization in larvae treated with each dose of peptide was observed compared to the control, where PBS was administered (data not shown).

**Figure 4:**
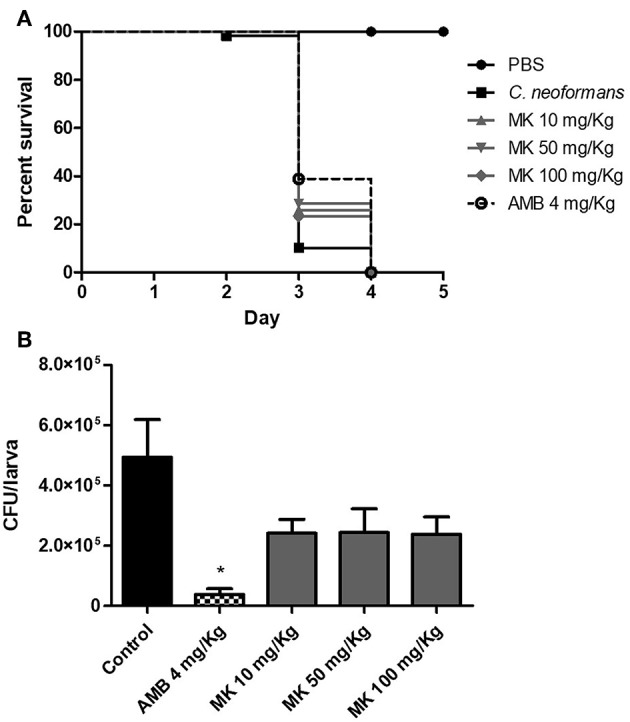
Effect of different doses of MK58911 (10, 50, and 100 mg/Kg) or amphotericin B (4 mg/Kg) on **(A)** survival and **(B)** fungal burden of *G. mellonella* larvae infected with 1 × 10^5^ cells/larvae. Six independent experiments were performed for survival curves, which resulted in a total of 48–60 larvae/group; three independent experiments were performed for fungal burden, which resulted in total of 15 larvae/group. ^*^p < 0.01 amphotericin B compared to control group.

Larvae infected with *C. neoformans* were treated with the same doses of MK58911 (10, 50, and 100 mg/Kg) to evaluate peptide efficacy. Larvae infected with 1 × 10^5^ cells/mL of fungus showed a high death rate, mainly occurring on the third day (Figure 4A). All concentrations of MK58911 tested had a similar therapeutic effect in the infected larvae, significantly delaying larvae mortality, with percentage of larvae survival to ~26, 29, and 23%, respectively, compared to the non-treated group (10%, *p* < 0.05) on the third day. When survival rates for the standard drug amphotericin B (4 mg/Kg) were measured, we observed a significant delay in larvae mortality, with percentage of larvae survival to ~39% (*p* < 0.01). We also verified the treatment efficacy for MK58911 given in combination with standard drugs. Since no difference in peptide response at the tested doses was observed in the *G. mellonella* model, we selected a dose of 10 mg/Kg to perform these experiments. The survival of larvae treated with the combination MK58911 10 mg/Kg + amphotericin B 4 mg/Kg or MK58911 10 mg/Kg + fluconazole 10 mg/Kg was not significantly different with that of larvae treated solely with amphotericin B or fluconazole.

Furthermore, we evaluated the effect of MK58911 on fungal burden in *G. mellonella* after 24 h of treatment. For PBS-treated larvae, the CFU increased after 24 h compared to administered inoculum (4.9 × 10^5^ vs. 1 × 10^5^ CFU/larva) (Figure 4B). Although not statistically significant, treatment of larvae with all doses of MK58911 showed a similar trend in decreasing the number of CFU/larva by ~2-fold compared to control group (2.4 × 10^5^ vs. 4.9 × 10^5^ CFU/larva). Amphotericin B significantly reduced the fungal burden by around 13-fold compared to the control group (3.7 × 10^4^ vs. 4.9 × 10^5^ CFU/larva, *p* < 0.01).

### MK58911 Did Not Have an Effect on Haemocyte Density

**Figure 5:**
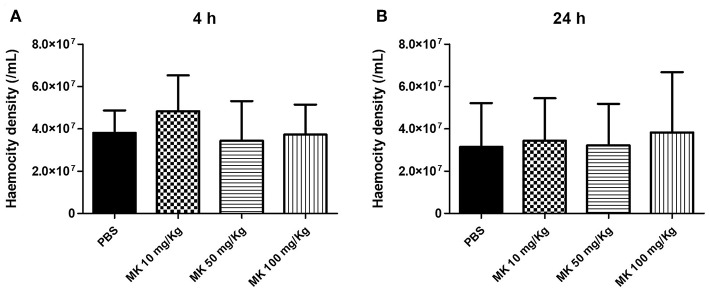
Effect of different doses of MK58911 (10, 50, and 100 mg/Kg) on haemocity density of *G. mellonella* larvae at **(A)** 4 h and **(B)** 24 h. Three independent experiments were performed for haemocity density, which resulted in total of 15 larvae/group.

To investigate whether administration of MK58911 could stimulate an immune response aside from its antifungal activity, the ability of this peptide to alter haemocyte density was determined after 4 or 24 h of treatment. MK58911 did not alter the number of haemocytes in the larvae hemolymph, and no significant differences were observed between haemocyte density in the peptide-treated and control groups for both evaluated time points (Figures 5A,B).

## Discussion

AMPs of the mastoparan class, isolated from wasp venom, have broad-spectrum activity with both antimicrobial and antitumor properties. Previous studies have demonstrated that this peptide class has activity against Gram-positive and Gram-negative bacteria (Souza et al., 2015). Another mastoparan peptide known as Polybia-MPII was active against *C. neoformans* with an EC_50_ of 17.7 μg/mL and EC_90_ of 36.6 μg/mL (Silva et al., 2017). Our results demonstrated that the peptide MK58911 exhibited activity against *Cryptococcus* and *Paracoccidioides* species, with MIC values ranging from 7.8 to 31.2 μg/mL (Table 1), denoting a potent antifungal activity.

The SI value is an important consideration in the development of new antimicrobial agents, since it evaluates the ratio between safety and potency. MK58911 was not toxic in lung and neural cells at tested concentrations and had an IC_50_ of >500 μg/mL for both mammalian cells (Table 1). Thus, this peptide demonstrated an effective SI value (>16). According to previous studies, a compound with an SI equal to or greater than 10 may be highly specific (Vicente et al., 2009; Ochoa-Pacheco et al., 2017).

We also tested a combination of two standard antifungals mixed at varying concentrations with the peptide MK58911. This methodology of combining antifungal agents presents several advantages, such as a greater spectrum of activity and potency and a reduction in toxicity, thus improving safety and possibly decreasing the development of resistance. The checkerboard method can be applied in the case where combining two compounds may result in a complementary mechanism of action (Lewis and Kontoyiannis, 2001; Mukherjee et al., 2005). MK58911 given in combination with amphotericin B or fluconazole demonstrated an indifferent interaction, suggesting that these antifungal drugs and MK58911 have similar targets and/or mechanisms of action. We also evaluated treatment combinations *in vivo* using the *G. mellonella* model. However, neither the MK58911 (10 mg/Kg) + amphotericin B (4 mg/Kg) combination nor the MK58911 (10 mg/Kg) + fluconazole (10 mg/Kg) combination had a synergic effect on larvae infected with *C. neoformans*.

Antifungal peptides work through a variety of mechanisms, such as inhibiting attachment to the external cell wall or membrane, forming pores in the lipid bilayer, or through receptor binding. In addition, other peptides lead to the production of reactive oxygen species and apoptosis through activation of signaling cascades or intracellular receptor binding (van der Weerden et al., 2013). A previous study showed that MK58911 interacts with a membrane-mimetic system (Souza et al., 2015). Thus, we decided to use a propidium iodide influx assay to investigate whether this action also occurs on the fungal cell membrane. When a compound is able to damage the membrane by altering its permeability, propidium iodide migrates into the cell and intercalates with the nucleotide bases of fungal deoxyribonucleic acid (DNA), turning the cell fluorescent (Choi and Lee, 2014).

When we tested the MIC as well as higher and lower concentrations (0.5 × MIC and 2 × MIC), we found that MK58911 has potent activity in the membrane integrity of *C. neoformans* (Figure 1). More activity occurred at 4 h when compared to 24 h, and the efficacy of this compound was higher than that of the drugs currently used for treating cryptococcosis (amphotericin B and fluconazole). In addition, MK58911 also showed concentration-dependent activity. Amphotericin B had the best effect at 4 h, since its mechanism of action involves binding directly to ergosterol and increasing membrane permeability, leading to rapid fungal death. On the other hand, fluconazole showed the highest membrane activity at 24 h, since its mechanism of action involves blocking ergosterol synthesis, a slower process (Odds et al., 2003; Scorzoni et al., 2017). MK58911 works rapidly and directly on the membrane, unlike the other antifungal drugs. We hypothesize that mastoparan peptides are interacting with phospholipids of cell membranes through the “carpet” mechanism, which leads to pore formation and, subsequently, to cytolysis (da Silva et al., 2014). However, further studies are necessary to investigate this hypothesis related to MK58911.

We also evaluated if the membrane damage caused by MK58911 exposure was due to fungal death by apoptosis or necrosis, which were measured by annexin V and PI staining. Annexin V has a high affinity for phosphatidylserine, a main component of the lipid bilayer inner surface. When apoptosis is initiated, a rapid exposure of phosphatidylserine to the outer bilayer surface occurs, which leads to recognition by annexin V. Additionally, an influx of propidium iodide occurs in cells undergoing necrosis or late apoptosis (Sharon et al., 2009). We could therefore distinguish apoptosis and necrosis through the use of annexin V and PI with flow cytometry (Crowley et al., 2016).

At 4 h, MK58911 appears to promote necrosis as the main effect, since these effects were significant and concentration dependent (Figure 2). At 24 h, an increase in apoptotic cells was observed compared to control. Similarly, amphotericin B induced necrotic events initially and apoptotic events later, which has also been seen in previous studies (Phillips et al., 2003; Mousavi and Robson, 2004). On the other hand, fluconazole caused a significant percentage of necrotic events compared to the control at 24 h, when the highest membrane damage was also observed.

The production of ROS is a known trigger in the cell death cascade. The pro-oxidant activity of ROS leads to an imbalance in stationary state of the cell, promoting so-called oxidative stress. This imbalance results in damage to biomolecules, such as DNA, lipids, proteins, and carbohydrates, which interfere with cell survival, death, and proliferation (Circu and Aw, 2010; Delattin et al., 2014). Thus, the increase of ROS in fungal cells may be responsible for a series of intracellular events that result in different types of cell death. In addition to promoting membrane damage, some antimicrobial peptides induce ROS accumulation, leading to fungal cell death (De Brucker et al., 2011; Hwang et al., 2011; Delattin et al., 2014). Because of this, we also investigated the action of MK58911 on ROS production. However, exposure to MK58911 for 4 h had no significant effect on oxidative stress in *C. neoformans* cells (Figure 3). In the same way, we observed no significant differences between fluconazole-treated cells and the control group, which is supported by a previous study (Ferreira et al., 2013). In contrast, amphotericin B exposure resulted in a concentration-dependent increase on the production of ROS. This event causes an initial oxidative burst and can also contribute to damage of the *C. neoformans* cell membrane through oxidation of membrane lipids (Sangalli-Leite et al., 2011).

Recently, *G. mellonella* have been used to investigate fungal pathogenesis as well as toxicity and efficacy of antifungal agents. These larvae have additional benefits compared to others invertebrate models, such as an incubation temperature of 37°C, which mimics infection conditions in mammals, and precise concentrations of pathogens and antimicrobial drugs can be administered to the prolegs (Fuchs and Mylonakis, 2006; Desalermos et al., 2012; Arvanitis et al., 2013). On the other hand, the use of *G. mellonella*, as with any model organism, has some drawbacks. Some systems/organs related to infection (adaptative immune system, lung, nervous central system) are absent or not easily comparable to those of mammals. In addition, the lack of standardized and mutant strains of *G. mellonella* may limit reproducibility of methods and dissemination of the model, but its recent genome sequencing can solve these problems (Binder et al., 2016; Lange et al., 2018).

In this study, MK58911 administered at doses of up to 100-fold the MIC did not result in toxic effects, such as death or melanization in *G. mellonella* larvae, which is similar to what was seen in lung and neural cells. Amphotericin at a concentration as high as 4 mg/Kg has been tested in the *G. mellonella* model with no toxicity, according to previous studies from our group (de Lacorte Singulani et al., 2016; Sangalli-Leite et al., 2016). In addition, MK58911 was effective in larvae infected with *C. neoformans*, and a significant delay in larvae mortality was observed with all doses of peptide treatment (*p* < 0.05, Figure 4). These effects were not dose-dependent, and a possible explanation is that the peptide is either being metabolized or is forming a complex with components of haemolymph, thus limiting activity at higher doses (Gibreel and Upton, 2013). However, low doses were sufficient for achieving an *in vivo* antifungal activity.

Several features of the innate immune response are conserved between insects and mammals. For instance, *G. mellonella* has six important cell types, or haemocytes, in its defense system. Specifically, haemocytes that are plasmatocytes or granulocytes have key roles in microbial defense, including phagocytosis, nodule formation, and encapsulation of microorganisms (Tojo et al., 2000). Thus, a fluctuation in haemocyte density in the hemolymph is observed based on the pathogenicity of the fungus (Bergin et al., 2003; Scorzoni et al., 2015; de Lacorte Singulani et al., 2016). In addition, some antifungals, such as lipopeptides, caspofungin, and micafungin cause a significant increase in the number of haemocytes in the *G. mellonella* model (Kelly and Kavanagh, 2011; Fuchs et al., 2015). We also evaluated if therapeutic effects of MK58911 on larvae could be related to a variation in haemocyte density. However, there were no significant differences between haemocyte density in the groups treated with each concentration of MK58911 and the control group (Figure 5).

## Conclusion

In conclusion, MK58911 is not toxic in two mammalian cells (lung fibroblasts and glioblastoma cells) and in the *G. mellonella* model and demonstrates both *in vitro* and *in vivo* antifungal efficacy. Moreover, the peptide acted on the membrane of fungal cells and induced necrosis. These results are promising, and further studies should determine whether similar results can be obtained in a murine model. Finally, MK58911 can be used as an example in the rational design of peptide analogs as a new class of antifungal agents.

## Data Availability Statement

The datasets generated for this study are included in the manuscript.

## Author Contributions

JS and MM contributed to the research idea and experimental design. JS, MG, MR, and CS performed the experiments. PG, BS, and MP synthesized the peptide. JS, MG, MR, and CS analyzed the data. JS, MG, and MR prepared and wrote the manuscript. PG, CS, MP, AF, and MM revised the final draft of manuscript.

### Conflict of Interest

The authors declare that the research was conducted in the absence of any commercial or financial relationships that could be construed as a potential conflict of interest.
